# The role of TNF-α in rheumatoid arthritis: a focus on regulatory T cells

**Published:** 2016-09-15

**Authors:** Mark Farrugia, Byron Baron

**Affiliations:** 1 *Center for Molecular Medicine and Biobanking, Faculty of Medicine and Surgery, University of Malta, Msida, Malta*

**Keywords:** rheumatoid arthritis, regulatory T cells, TNF-α therapy, joint inflammation

## Abstract

The autoimmune disorder rheumatoid arthritis (RA) causes chronic inflammation and destruction of joints. T cells are a predominant component of the synovial environment in RA, however the functional role of these cells is not yet fully understood. This is in part due to the fact that the balance and importance of the relation of T_regs_ with T-effector cells in RA is still under investigation. The current treatment regimen for this debilitating disease focuses on controlling symptoms and preventing further joint damage through the use of therapies which affect different areas of the immune system at the synovium. One of the main therapies involves Tumor Necrosis Factor alpha (TNF-α) inhibitors. In the RA immune-environment, TNF-α has been shown to have an influential and extensive but as yet poorly understood effect on T_reg_ function in vivo, and undoubtably an important role in the treatment of RA. Interestingly, the high levels of TNF-α found in RA patients appear to interfere with the mechanisms controlling the suppressive function of T_regs_.

**Relevance for patients:** This review focuses on the conflicting literature available regarding the role played by T_regs_ in RA and the impact of TNF-α and anti-TNF-α therapies on T_regs_ in this scenario. Individuals suffering from RA can benefit from better insight of the treatment mechanisms of the immunologic processes which occur throughout this disease, as current treatments for RA focus on several different areas of the immune system at the synovial compartment.

## 1. Introduction

Rheumatoid arthritis (RA) is an autoimmune disorder that manifests itself as a chronic inflammation of the lining of the joints, with significant morbidity and mortality rates if left untreated [1]. RA is characterized by synovial inflammation and hyperplasia (swelling), autoantibody production (rheumatoid factor (RF) and anti-citrullinated protein antibody (AC-PA)), cartilage and bone destruction, and systemic features, including cardiovascular, pulmonary, psychological, and skeletal disorders [2]. Possible risk factors for the development of RA include genetic background, smoking, silica inhalation and periodontal disease [1].

A hyperplastic synovium is the major contributor to the cartilage damage in RA. The loss of the protective effects of the synovium result in the alteration of the protein-binding characteristics of the cartilage surface, promoting synoviocytes (FLS) adhesion and invasion. These processes lead to the destruction of the surface [2]. Bone erosion then follows rapidly (affecting 80% of patients within 1 year after diagnosis [1]). Cytokines present in the synovial fluid, particularly macrophage colony-stimulating factor (M-CSF) and receptor activator of NF-κB ligand (RANKL), promote osteoclast differentiation and invasion of the periosteal surface adjacent to articular cartilage [3]. Tumor Necrosis Factor alpha (TNF-α) and Interleukin (IL) -1, 6, and potentially 17 amplify osteoclast differentiation and activation.

Studies in Europe have shown that there is a gradient in the prevalence of RA, starting from a low prevalence in the South (e.g. Italy 0.31%) [4], to a higher prevalence in the North (e.g. Finland 0.8%) [5]. While no formal epidemiológica! studies on RA have been carried out in Malta yet, a total of approximately 600 patients with the disease are followed up at the Rheumatology Clinic at St. Luke’s Hospital, giving a prevalence of 0.16%[6].

The use of different case definitions makes the estimates vary as widely as 25 to 115 per 100,000 [7]. The annual incidence rate of RA recorded in studies varies between 20 and 50 cases per 100,000 in Northern European countries, but there are indications that it may be lower in Southern European countries [8,9]. Studies of the incidence and prevalence of RA suggest variations between different populations even within the same country. Possible explanations include regional variation in behavioral factors, climate, environmental exposures, RA diagnosis, and genetic factors [7].

**Figure 1.**
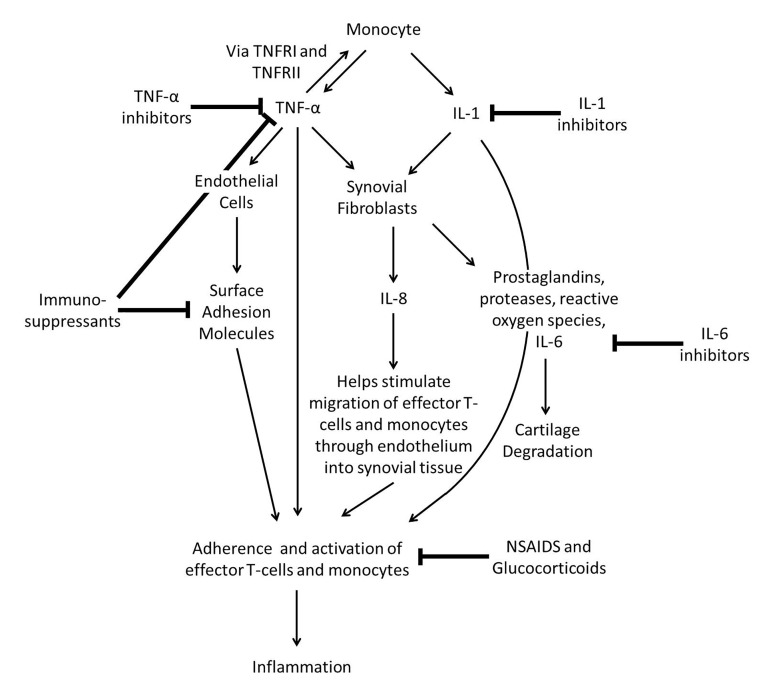
Summary of therapies for rheumatoid arthritis. Treatment regimens for RA have been generally divided into two. The first category contains the non-steroidal anti-inflammatory drugs (NSAIDs) and glucocorticoids, which block effector T-cell activation and reduce inflammation. Other therapies such as immunosuppressants, steroids and biological therapies are grouped under the non-specific term of disease-modifying anti-rheumatic drugs (DMARDs) and these include: Tumor Necrosis Factor alpha (TNF-α) inhibitors (which diretly block TNF-α), Interleukin (IL-1 and IL-6) inhibitors (which target cartilage destruction via synovial fibroblast modulation), immune-suppressants (which inhibit surface adhesion molecules), T-cell activation inhibitors (that bind to differentiated T cells, reducing overall T-cell numbers), B-cell depletors (which act specifically by targetting and destroying B-cells) amd Janus kinase (JAK) inhibitors (blocking signal transduction of cytokine receptors).

Currently, treatment focuses on controlling symptoms and preventing further joint damage. Medications used in the treatment of RA include non-steroidal anti-inflammatory drug (NSAIDs), disease-modifying anti-rheumatic drug (DMARDs), TNF-α inhibitors, IL-6 inhibitors, T-cell activation inhibitors, B-cell depletors, Janus kinase (JAK) inhibitors, and steroids [10] (Figure 1). Since all current treatments for RA are focusing on different areas of the immune system at the synovial compartment, a good understanding of the immunologic processes which occur throughout RA is vital for a better insight of the treatment mechanisms themselves.

## 2. Synovial immunological processes

Several of the risk alíeles linked to RA consistently map functionally with immune regulation such as the nuclear factor kappa-light-chain-enhancer of activated B-cells (NF-κB)-dependent signaling, T-cell stimulation, activation, and functional differentiation. This suggests that these immunologic pathways are amongst the key modulators of the development of the autoimmune inflammation in RA [11-13].

**Figure 2.**
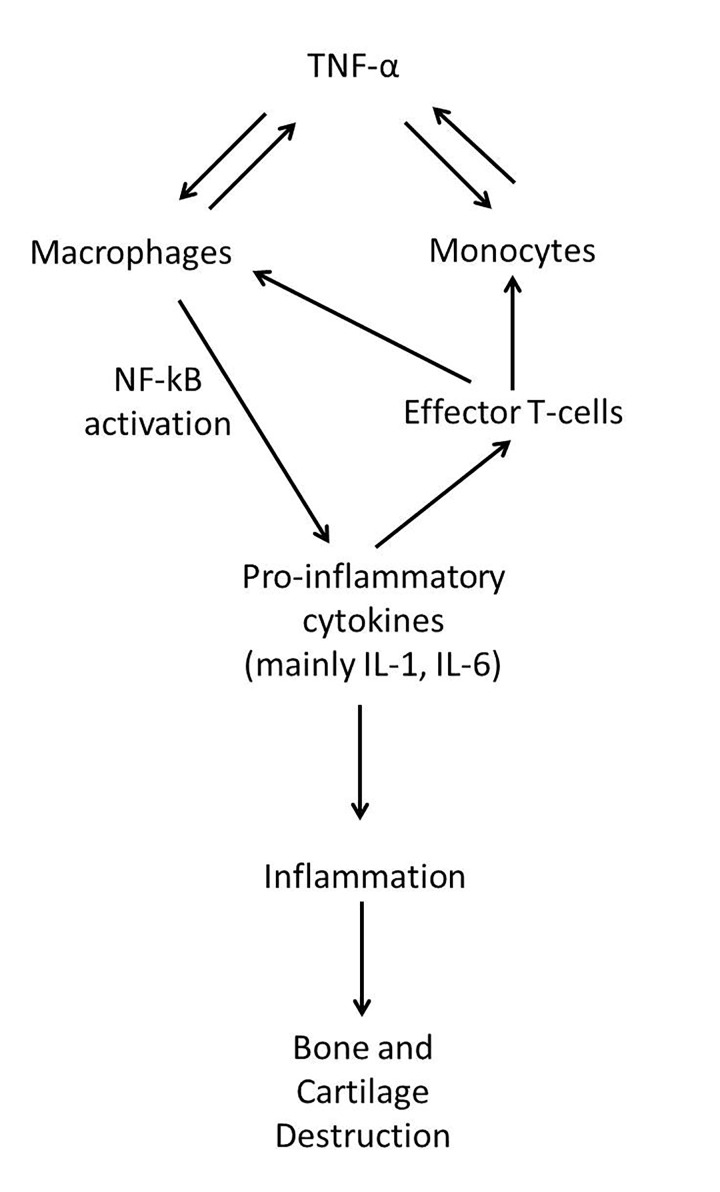
The role of TNF-α in rheumatoid arthritis. Immune regulation is at the heart of RA with the generating of an autoimmune response, with TNF-α being a major player. Dendritic cells, T cells, and B-cells are costimulated, and this leads to T-cell activation and functional differentiation. The stimulated macrophages in turn activate nuclear factor kappa-light-chain-enhancer of activated B-cells (NF-κB)-dependent signaling, which induces pro-inflammatory cytokines that enhance local inflammation of the synovial membrane (synovitis) and result in damage to cartilage and bones.

The costimulation-dependent interactions among dendritic cells, T cells, and B-cells are thought to occur primarily in the lymph node, generating an autoimmune response to citrulline-containing self-proteins [2]. The inflammation of the synovial membrane (synovitis) is then caused by the infiltration of leukocytes in the synovial compartment. Leukocyte migration is enabled through various pathways, mainly through the activation of endothelial tissue in synovial micro-vessels, resulting in an increase in expression of adhesion molecules and chemokines [14]. This and other processes result in the build-up of inflammatory synovial tissue (Figure 2).

A variety of innate effector cells, including macrophages, mast cells, and natural killer cells, are found in the synovial membrane, while neutrophils reside mainly in synovial fluid. The main role of macrophages in this scenario is that of releasing cytokines (e.g., TNF-α and interleukin-1, 6, 12, 15, 18, and 23), reactive oxygen intermediates, nitrogen intermediates, production of prostanoids and matrix-degrading enzymes, phagocytosis, and antigen presentation [15]. Neutrophils on the other hand contribute to synovitis by synthesising prostglandins, proteases, and reactive oxygen intermediates [16]. These findings provide evidence that actiation of the innate immune pathway contributes to synovitis.

It has become more apparent from various reports in the literature that cytokines play an integral role in the activation and maintanence of the innate immune pathway. Cytokine production that arises from numerous synovial cell populations is central to the pathogenesis of RA [13]. TNF-α is one such cytokine and plays a fundamental role through the activation of cytokine and chemokine expression, expression of endot-helial-cell adhesion molecules, protection of synovial fibro-blasts, promotion of angiogenesis, suppression of regulatory T cells, and induction of pain [17,18]. The central role of this cytokine has been repeatedly confirmed by a successful therapeutic blockade of membrane and soluble TNF-α in patients with RA.

## 3. TNF-α

TNF-α is an inflammatory cytokine consisting of a trimeric protein encoded within the major histocompatibility complex. It’s first identified form was the 17 kDa secreted form, but further research then showed that a noncleaved 27 kDa precursor form was also present in transmembrane form [19]. TNF-α and its specific receptors TNFR1/TNFR2 are the major members of a gene superfamily of ligand and receptors which are respondible in regulating essential biologic functions. The extracellular domains of TNFR1 and TNFR2 are homologous and have similar affinity for TNF-α, however the cytoplasmic regions of these two receptors are distinct and mediate different downstream events. TNFR1 signalling is the major mechanistic pathway responsible for the effects of TNF-α [20]. These receptors are expressed on all somatic cells.

## 4. Role of T cells in rheumatoid arthritis

Even though T cells are a predominant component of the synovial environment in RA, the functional role of T cells is not yet fully understood. This is mainly due to the fact that lymphocytes, including T cells, act and react according to the presence and numbers of other subsets of lymphocytes, and this systematic approach to immunity has only recently started to be investigated in detail [21]. Therefore, it is vital to understand all of the main protagonists in the synovium in our assessment of the immunological processes taking place.

Activated CD4^+^ T cells stimulate monocytes, macrophages, and synovial fibroblasts to produce the cytokines IL-1, IL-6, and TNF-α [22]. Activated CD4^+^ T cells also stimulate B-cells and these produce immunoglobulins, including the RF. The precise immunologic role of RF is still unknown, but it may involve the activation of complement through the formation of immune complexes [23]. Activated CD4^+^ T cells also express RANKL, and as explained previously, this stimulates osteoclast differentiation. Thus these activated T cells give rise to cartilage erosion caused by excessive osteoclasts [24].

RA is conventionally considered to be a disease mediated by type 1 helper T cells, however there is increased attention on the role of type 17 helper T cells (Thl7). Thl7 is a subset of T cells that produces interleukin-17A, 17F, 21, and 22 and TNF-α [25,26]. Other cytokines which support the differentiation of Thl7 cells are macrophage-derived and dendritic cell-derived transforming growth factor β (TGF-β) and IL-1, 6, 21, and 23 [26].

It is interesting to note that IL-6 suppresses the differentiation of regulatory T cells (T_regs_), thus shifting T-cell homeostasis toward inflammation rather than autoregulation [27]. It is now well accepted that T_regs_ are critically involved in immune tolerance and homeostasis. T_regs_ that are detected in tissues from patients with RA seem to have limited functional capability, as inferred via Forkhead box P3 (FoxP3) transcript levels, which are lower in the synovial membrane compared to those in peripheral blood or synovial fluid [28].

In RA, there are two distinct classes of T_regs_ depending on their location: those found in the peripheral blood and those at the site of inflammation, usually studied in the synovial fluid (SF) [29-31]. Different studies report different accumulation numbers of T_regs_ in the peripheral blood between healthy individuals and RA patients, varying from reports of decrease to an increase in T_regs_, comparatively [32-36]. In many scenarios however, the lack of function of the T_regs_ themselves seems to be observed. FoxP3^+^ T_regs_ sampled from the SF of RA patients are able to suppress the proliferation of effector T cells [31], but Ehrenstein et al. reported that, while T_regs_ from RA patients do suppress proliferation, they are defective in their ability to suppress pro-inflammatory cytokine production [34], and thus this process is not regulated, resulting in inflammation. It is important to note that this study was done with T_regs_ obtained from peripheral blood rather than SF. On the other hand, a conflicting recent study also performed using T_regs_ obtained from peripheral blood, shows that there is no significant difference between the suppressive effects of FoxP3^+^ T_regs_ on certain cytokines and the proliferation of effector T cells, between T_regs_ obtained from healthy individuals and from RA patients [37].

Various studies provide compelling evidence that CD4^+^ FoxP3^+^ T_regs_ cells play an indispensable role in maintaining immune homeostasis and in suppressing deleterious excessive immune responses [38]. There are various subsets of T_regs_, with various effects on effector T cells in the autoimmune scenario [39]. Any disregulation or loss of function in T_regs_ will result in an upregulation of T-effector cells and any other cell type under suppression by T_regs_ Thus it is important to understand better how TNF-α, being one of the most present and influential cytokines in the synovial immuno-environment, affects the function of T_regs_.

## 5. Effect of TNF-α on regulatory T-cell function in rheumatoid arthritis

TNF-α and IL-7 are two cytokines which act against the suppressive activity of human T_regs_ [40]. High levels of TNF-α are found in both the serum and synovial fluid of RA patients, and therefore this might be one of the factors which result in defective T_regs_ function [41]. Treatment of these patients with infliximab, an anti-TNF-α therapy, gave rise to an adaptive FoxP3^+^ CD62L~ T_regs_ population, which was able to suppress cytokine production of effector T cells via a TGF-β and IL-10 pathway [42]. The fact that the naturally-occurring CD62L^+^ T_regs_ remained defective in infliximab-treated patients clearly showed that TNF-α was responsible in promoting the development of a new dysfunctional subset of FoxP3^+^ T_regs_. Another study highlighting the importance of TNF-α in RA vis-á-vis FoxP3^+^ T_regs_ reported that overexpression of TNF-α in human TNF-α transgenic mice led to the development of arthritis, with an increased number of T_regs_ expressing the TNF receptor II (TNFRII) [43]. TNFRII is a required receptor for TNF-α interactions [44]. The TNF-α overexpression did not inhibit the suppressive activity of the T_regs_, however the T_regs_ still failed to control inflammation. When TNF-α was blocked, a further increase in the frequency of T_regs_ was observed, and these T_regs_ had upregulated CTLA-4 expression, resulting in enhanced suppressor activity [43]. The TNF-α also induced the differentiation of a CD62L^-^ Treg population as observed in the previous study [42].

These studies suggest that TNF-α plays an important role in the inhibition of FoxP3^+^ T_reg_ suppressive function, particularly in suppressing inflammation. Further to this, it has been shown that TNF-α signaling via TNFRII downregulates FoxP3 expression in humans in both naturally-occurring T_regs_ and adaptive Tregs, and this results in the inhibition of T_reg_ suppressive activity [45]. Another study done using human cell cultures showed that during the inhibition of active T_regs_ via TNF-α signaling of the TNFRII receptor, the TNF-α activated the canonical NF-κB pathway and induced a pro-inflammatory phenotype [46], however in this case FoxP3 expression was not affected. The inhibition of T_reg_ suppressive activity could be reversed by treatment with anti-TNFRII antibody. This shows that TNF-α signaling via TNFRII may be one mechanism which leads to T_reg_ defects in RA. This study was performed using both T_regs_ from peripheral blood and synovial fluid and the results showed similar trends in both types of T_regs_.

Another way TNF-α might inhibit T_reg_ suppressive activity is by influencing the formation of the immunological synapse between T_regs_ and antigen presenting cells (APCs) [47]. Although for effector T cells, protein kinase C-θ (PKC-θ) recruitment to the immunological synapse is necessary for full T-cell activation, for FoxP3^+^ T_regs_, PKC-9 is concealed from the immunological synapse. A study was conducted in which a model system on supported planar bilayers containing the mobile fluorescently labelled intercellular adhesion molecule-1 (ICAM-1), anti-CD3 antibodies and CD4+CD25+ effector T cells or CD4^+^ CD25^+^ T_regs_ (freshly isolated from peripheral blood) was devised, in order to emulate the immunological synapse. Addition of TNF-α to this model increased the PKC-9 recruitment to the T_reg_ immunological synapse, and this inhibited their suppressive activity. This contrasts with the fact that after blocking PKC-θ, T_reg_ function was enhanced [47]. This study showed that inhibition of PKC-θ might protect T_regs_ from inactivation by TNF-α and this restores the suppressive function of defective T_regs_ in RA patients. Disc large homolog 1 (Dlghl) is another identified molecule involved in the immunological synapse formation which regulates T_reg_ function independently of PKC-θ [48]. Dlghl was found to be recruited to the immunological synapse four times as much in T_regs_ than in effector T cells. It was also found that T_regs_ from RA patients with active disease had defective Dlghl recruitment to the immunological synapse. This defective recruitment resulted in reduced suppressive activity of T_regs_. Exposing healthy control Tregs to TNF-α decreased the Dlghl recruitment and thus also reduced T_reg_ suppressive activity [48]. These findings suggest that in FoxP3^+^ T_regs_, PKC-θ-mediated negative and Dlghl-mediated positive pathways seem to regulate suppressive function independently, and in RA, one or both of these pathways may be defective as a possible con4equence of TNF-α [49].

TNF-α was also found to interact directly with the DNA-binding activity of the *FOXP3* gene in T_regs_ [50]. In this analysis done on human cells obtained from both the peripheral blood and the synovial fluid of RA patients it was shown that TNF-α - TNF receptor-binding induces increased expression of protein phosphatase 1 (PP1). PP1 dephosphorylates Ser418 in the DNA-binding domain of the FoxP3 transcription factor, and this in turn reduces its DNA-binding activity, thus impairing the suppressive function of T_regs_.

**Figure 3.**
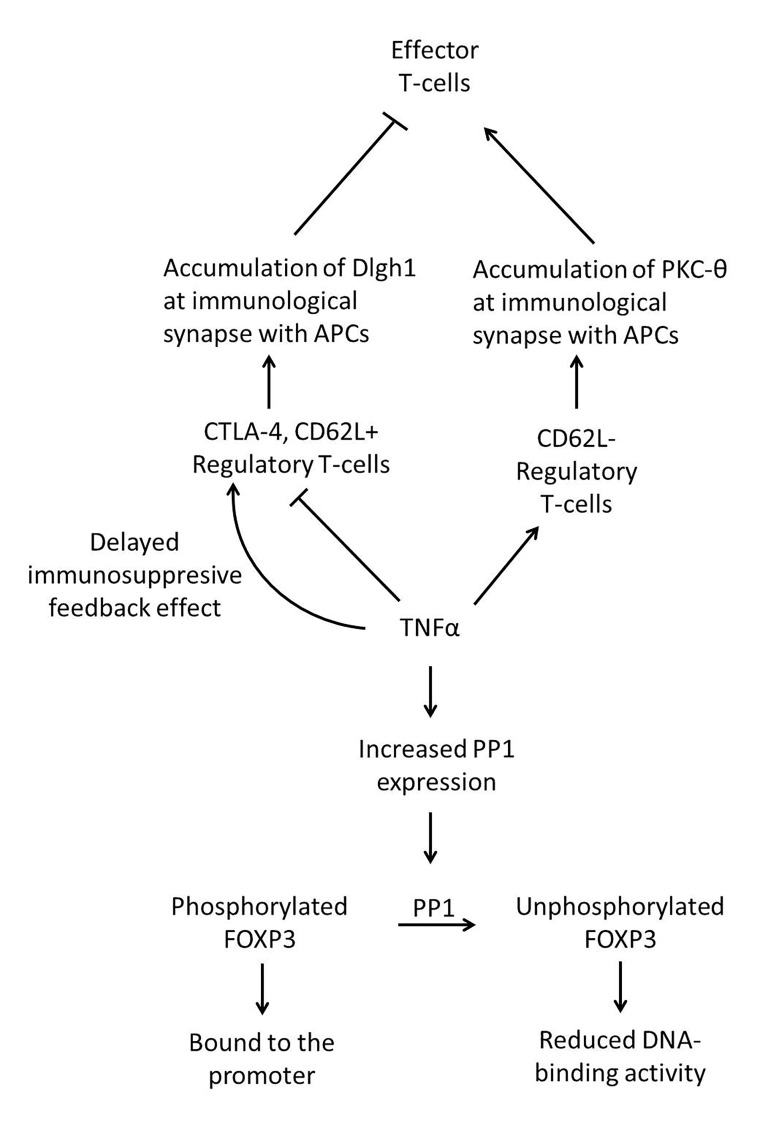
The biochemistry of rheumatoid arthritis focusing On T_regs_. In RA patients high levels of TNF-α counteract the suppressive activity of human T_regs_, acting as a factor towards defective T_reg_ function. One mechanism is through increased protein phosphatase 1 (PP1) expression, which physically interacts with the DNA-binding domain of the FoxP3 transcription factor and dephosphorylates Ser418, leading to decreased DNA-binding activity. A second mechanism is through increased protein kinase C-9 (PKC-9) recruitment to the T_reg_ immunological synapse. Yet another mechanism is via reduced recruitment of disc large homolog 1 (Dlghl) to the immunological synapse. Either or all of these can act in RA patients to reduce T_reg_ suppressive function.

Interestingly, although TNF-α is a major pro-inflammatory cytokine, there is increasing evidence that indicates TNF-α also has immunosuppressive feedback effects, as was demonstrated in a study where both resting and activated mouse peripheral FoxP3^+^ T_regs_ purified from lymph node expressed remarkably higher surface levels of TNFRII than effector T cells in vitro [51]. In the same study it was observed that in co-cultures of Tregs and effector T cells, suppression of effector T-cell proliferation by T_regs_ was initially observed after exposure to TNF-α, however longer exposure to TNF-α restored the suppressive effects. Furthermore, TNF-α expanded T_reg_ populations in this study, and these TNF-α-expanded T_regs_ had up-regulated expression of CD25 and FoxP3, enhancing the suppressive activity of these T_regs_. Thus in this study the stimulatory effect of TNF-α on T_regs_ resembled the reported costimulatory effects of TNF-α on effector T cells. Another study to determine the effect of TNF-α on T_regs_ showed that when T_reg_ cells were cultured for 20 h with or without IL-2 before the suppression assays, the presence of TNF in the pre-culture had no effect on their suppressive function in any assay condition [52]. This work also showed that in the presence of IL-2, the effects of TNF on human T_regs_ in a 3-day culture of whole CD4^+^ T cells resulted in an increased proportion of T_regs_ and the upregulation of FOXP3 expression. A suggestion for the slower response of Tregs to TNF-α could be a delayed immunosuppressive feedback effect [51]. Another study concludes that human Tregs obtained from the buffy coat of healthy donors which were deficient in TNFRII were not able to control inflammatory responses in vivo [53]. TNFRII expression on human T_regs_ present in the synovial fluid of RA patients is also up-regulated [45], presumably reflecting their enhanced suppressive capacity [33]. It is not clear whether TNFRH^+^ FoxP3^+^ T_regs_ are more functionally suppressive (Figure 3).

Diverse roles of TNF-α in the immune response may be partly explained by the existence of two forms of this cytokine: a membrane-bound TNF-α (mTNF-α), and TNF-α which is cleaved from the membrane and released as a soluble cytokine (sTNF-α) [54]. Moreover, TNFRI and TNFRII have different expression patterns and affinities for mTNF-α or sTNF-α, and may transduce signals with opposite outcomes. TNFRII binds with higher affinity to mTNF-α than to sTNF-α [55].

## 6. anti-TNF-α therapy effects and future work

It is clear from the conflicting literature above, that we are still far from deducing the exact role and effect of TNF-α on T_regs_ in the RA scenario. TNF-α seems to interfere with the mechanisms controlling T_reg_ suppressive function, and therefore it is plausible to predict that anti-TNF-α therapies would counter this effect. In fact, studies show that anti-TNF-α therapy has a regulatory effect on the immune system of RA patients by promoting an increase in the proportion of T_regs_ and suppressing effector T cells [56]. One such recent study showed that the anti-TNF antibody adalimumab promoted the interaction between monocytes and T_regs_ from RA patients by binding to monocyte membrane bound TNF, enhancing its expression and its binding to TNF-RII expressed on T_regs_ [57]. This resulted in adalimumab-expanded functional FoxP3^+^ T_regs_ able to suppress Thl7 cells through an IL-2/STAT5-dependent mechanism. This study demonstrated that a therapeutic antibody thought to act by blocking TNF-α can also enhance the regulatory properties of this pro-inflammatory cytokine.

However, clinical trials have accumulated evidence that anti-TNF-α therapies might promote rather than suppress certain forms of autoimmunity. In RA, anti-TNF-α therapy is sometimes associated with adverse events, such as multiple sclerosis and lupus [58]. Cases of juvenile arthritis patients who developed type 1 diabetes have been reported during therapy with a TNF-α antagonist [59,60].

TNF-α without a doubt has an important role in the treatment of RA, and it has been shown to have a powerful, varied and yet poorly understood effect on T_reg_ function in vivo in the RA immune-environment. Although anti-TNF-α therapies have been widely used to treat RA with significant clinical result, more research is still needed to understand better the total effect of such therapies on all cell types involved in the synovial immune-environment. Anti-TNF-α therapies might exhibit serious side effects, and the mechanisms leading to such side effects can be investigated further to find methods of suppressing them. Alternate routes to suppress the over-reactive effector T cells as well as activate and enhance the T_reg_ subsets can be investigated, whilst working to obtain a broader and clearer picture of the effect TNF-α has on T_regs_ and RA in general.

## Disclosures

The authors declare no conflict of interest.
